# Healthy babies through infant-centered feeding protocol: an intervention targeting early childhood obesity in vulnerable populations

**DOI:** 10.1186/1471-2458-11-868

**Published:** 2011-11-15

**Authors:** Mildred A Horodynski, Beth Olson, Susan Baker, Holly Brophy-Herb, Garry Auld, Laurie Van Egeren, Joel Lindau, Lisa Singleterry

**Affiliations:** 1 College of Nursing, Michigan State University, B 515 G West Fee Hall, East Lansing, MI 48824 USA; 2 Dept. of Food Science and Human Nutrition, Michigan State University, 2112 Anthony Hall East Lansing, MI 48824 USA; 3 Dept. of Food Science and Human Nutrition, Colorado State University, 102 E. Gifford Building, campus box 1571, Fort Collins, CO 80523-1571 USA; 4 Dept. of Family and Child Ecology, 3F Human Ecology, Michigan State University, East Lansing, MI 48824 USA; 5 Dept. of Food Science and Human Nutrition, Colorado State University, 105 E. Gifford Building, campus box 1571, Fort Collins, CO 80523-1571 USA; 6 University Outreach and Engagement, Michigan State University, 93 Kellogg Center, East Lansing, MI 48824 USA

## Abstract

**Background:**

Poor feeding practices during infancy contribute to obesity risk. As infants transition from human milk and/or formula-based diets to solid foods, these practices interfere with infant feeding self-regulation and healthy growth patterns. Compared with other socioeconomic groups, lower-income mothers are more likely to experience difficulty feeding their infants. This may include misinterpreting feeding cues and using less-than-optimal feeding styles and practices, such as pressuring infants during mealtimes and prematurely introducing solid food and sweetened beverages. The Healthy Babies trial aims to determine the efficacy of a community-based randomized controlled trial of an in-home intervention with economically and educationally disadvantaged mother-infant dyads. The educational intervention is being conducted during the infant's first 6 months of life to promote healthy transition to solids during their first year and is based on the theory of planned behavior.

**Methods/Design:**

We will describe our study protocol for a multisite randomized control trial being conducted in Colorado and Michigan with an anticipated sample of 372 economically and educationally disadvantaged African American, Hispanic, and Caucasian mothers with infants. Participants are being recruited by county community agency staff. Participants are randomly assigned to the intervention or the control group. The intervention consists of six in-home visits by a trained paraprofessional instructor followed by three reinforcement telephone contacts when the baby is 6, 8, and 10 months old. Main maternal outcomes include a) maternal responsiveness, b) feeding style, and c) feeding practices. Main infant outcome is infant growth pattern. All measures occur at baseline and when the infant is 6 and 12 months old.

**Discussion:**

If this project is successful, the expected outcomes will address whether the home-based early nutrition education intervention is effective in helping mothers develop healthy infant feeding practices that contribute to improving infant health and development and reducing the risk of early-onset childhood obesity.

**Trial Registration:**

Current Controlled Trials ACTRN126100000415000

## Background

The Institute of Medicine and Healthy People 2010 [1] identify childhood obesity as a serious nationwide health problem requiring urgent attention in the United States. An estimated 10 million children are overweight, a number that has doubled in the past 20 years [2], with the prevalence of overweight in infancy increasing from 11% to 17% [3]. Obesity is more prevalent in lower-income and minority children, placing them at increased risk for later health problems, such as hypertension, cardiovascular disease, and type II diabetes [4].

Although the causes of obesity among children are multilayered, data suggest that by toddlerhood, many children are at risk because of overeating. Thus, preventive feeding interventions are especially necessary during age-appropriate transition to solid foods. Mothers' misinterpretation of infant feeding cues, the use of controlling feeding styles, and poor feeding practices can affect infants' ability to regulate food intake [5]. Although mothers have some knowledge about infant nutrition and feeding cues as infants transition from human milk/formula-based diets to solid foods, they continue to rely on inappropriate indicators of satiation, such as infants' sleeping through the night and advice from extended family members [6]. Our study focuses on the promotion of healthy infant feeding practices in reducing the risk of early onset of childhood obesity.

Learning to feed effectively early on has been shown to help mothers develop positive feeding relationships with their infants and promote infant feeding self-regulation and normative growth patterns. Maternal responsiveness, maternal feeding style, and appropriate feeding practices are three critical components in the development of infant feeding self-regulation [7]. From a prevention perspective, maternal relationship and skill-building intervention approaches that target maternal responsiveness, feeding styles, and feeding practices must be evaluated for feasibility, effectiveness, and sustainability to reduce the risk of childhood obesity. To date, we have identified only one intervention study [8] designed to improve feeding behaviors of infants transitioning from human milk or formula to solid foods delivered by trained community nurses.

The Healthy Babies (HB) intervention we have designed addresses three major gaps that exist in infant feeding intervention research. First, few studies have been tailored to assist mothers of lower incomes with transitioning their infants to solid foods. Second, studies have historically focused on the feeding of preschoolers and older children rather than infants. Third, most existing interventions use models focused on nutritional knowledge enhancement without a relationship-skill component. Increasing maternal knowledge without addressing the family and cultural context and practices through which mothers apply this knowledge does not provide mothers with the relationship skills and strategies to change and improve maternal responsiveness, feeding styles, and feeding practices.

### Aims and Hypotheses

The aim of the study is to compare the effect of a home-based intervention for economically and educationally disadvantaged mothers of infants, 4 months of age or younger, versus standard nutrition education on maternal responsiveness, feeding style, and feeding practices.

Mothers with infants under 4 months of age will receive one of the two following interventions:

1) In- home HB intervention delivered by a trained paraprofessional instructor [a person who is appropriately trained and supervised in providing nutrition education instruction to families] prior to when the infant is 6 months of age promoting healthy feeding practices and enhanced mother-infant interaction, plus three reinforcing telephone contacts when the baby is 6, 8, and 10 months old.

2) Traditional nutrition education (EFNEP, the Expanded Food and Nutrition Education Program, a standard nutrition education curriculum offered in each state through county Cooperative Extension Service programs) consisting of six in-home nutrition education lessons delivered by a paraprofessional instructor prior to when the infant is 6 months of age that does not include extensive content on infant-centered feeding.

We hypothesize that the in-home HB intervention relative to usual nutrition education will be associated with:

• An increase in maternal responsiveness

• An increase in healthy maternal feeding style

• An increase in healthy age-appropriate infant feeding practices

• Healthy, age-appropriate infant growth patterns of weight and length

• Mothers' satisfaction with the HB intervention

• Feasibility in a community-based setting in the United States

## Methods/Design

### Overall study design

The design of this study is a randomized, experimental, short-term, longitudinal controlled trial (RCT) with a convenience sample of economically and educationally disadvantaged mothers of infants living in Colorado and Michigan. Participants are randomly assigned to the HB intervention or a control group with a goal of N = 139 participants per group by Time 3 data collection. There are three assessment periods: baseline (Time 1), when the infant is 6 months of age (Time 2), and when the infant is 12 months of age (Time 3). The United States Department of Agriculture (USDA) has funded this project for 3 years, making this multi-state collaboration between Michigan and Colorado possible.

### Development of the intervention

The HB intervention was developed and refined based on a pilot project, the Infant Feeding Series (TIFS) [9,10], that provided initial evidence of lower-income mothers delaying the early introduction of solid foods until the infant was between 4 and 6 months of age as recommended by the American Academy of Pediatrics. HB addresses core infant nutrition concepts but also focuses on: a) infant normative growth and development (e.g., infant cues, temperament, behavioral states); b) mother-infant feeding relationship skill-building (i.e., maternal responsiveness); support for mothers in creating their own infant feeding plans); and d) skills and strategies for implementing and sustaining their feeding plans and new feeding practices and styles. The curriculum focuses on a specific concern that emerged from our previous work: that economically and educationally disadvantaged mothers need help in delaying the early introduction of solids to their infants [10]. The Theory of Planned Behavior (TPB) [11] guided our understanding of maternal behaviors in the parent-infant feeding interactions and decisions. The TPB is commonly used to predict health-related behavioral intentions and behaviors. Organizing principles of the TPB provided the basis for the development of the HB lessons and the following framework for their delivery [12]. Staff members in Cooperative Extension Service programs in both states reviewed the lessons for appropriateness and feasibility.

### Participants and recruitment

Participants with the following characteristics are being recruited: low-income (≤ 185% federal poverty level in the US and eligible for federal food assistance programs); 18 years of age or over; mothers of infants less than 4 months of age who have not started solid foods; a primary caregiver of the infant, and neither mother nor infant has a diagnosed eating, physical, or chronic health problem. Mother-infant dyads are targeted for recruitment from local community programs, such as the Special Food Supplemental Nutrition Program for Women, Infants, and Children (WIC), a federal assistance program of the Food and Nutrition Service of the USDA, food pantries, hospitals, physicians' offices, and immunization clinics. Staff from these programs provide information about the study and encourage mothers with infants to enroll in the project.

Once eligibility is established, families are contacted for the initial data collection home visit where written informed consent is obtained. Newly recruited participants are not previously enrolled in EFNEP. We have obtained ethical approval to conduct this trial from the University Committee on Research Involving Human Subjects from Michigan State University and the Institutional Review Board for Human Subjects from Colorado State University. Informed consent for voluntary participation is obtained prior to commencement in the study.

### Randomization

Following consent and baseline data collection, mothers are randomized into either the HB or control group. This is done via computer minimization procedure, balancing groups with respect to the county recruitment locations. According to CONSORT guidelines [13], allocation concealment is used to prevent participants, data collectors, and paraprofessionals from knowing which group the participants will be assigned. Only after enrollment in the program do researchers assign participants into intervention or control groups. The assignment of groups is withheld from data collectors throughout the entire length of the study. Separate paraprofessional instructors deliver either the HB intervention or the traditional nutrition education; separate individuals who are not paraprofessional instructors are trained as data collectors in each county to do the in-home data collection at each time period.

### Intervention group

The Healthy Babies intervention consists of six in-home lessons taught by a trained paraprofessional instructor followed by three reinforcement telephone calls. Each lesson (60 minutes in length) is designed to promote the development of healthy infant eating and incorporates research-based information, opportunities for mothers to develop and practice skills, and a discussion of strategies to overcome challenges and problem-solving techniques. The three telephone contacts, about 5 minutes in length, made at 6, 8, and 10 months, are used to reinforce key concepts in the lessons after conclusion of the intervention to maintain effects and sustain contacts with participants to improve retention.

Six lessons have been identified as the number of lessons that best balance likelihood of completion by participants and likelihood of intervention impact. Six lessons have been found to produce behavioral change [14] and represent sufficient time to build a supportive relationship with the individual mothers.

During the six lessons, mothers will engage in activities in support of lesson goals. Intervention strategies focusing on feeding practices will include modification of the feeding environment (e.g., turning the TV off during feeding). An animated DVD, which includes actual footage of infant and parent behaviors, will be used to demonstrate infant temperament traits.

### Control group

The control group families receive the usual care provided by ENFEP. These families are newly enrolled into EFNEP as part of the HB study and have not received home visitation previously. The control lessons are delivered in a similar manner as the HB lessons, such that a paraprofessional instructor provides six lessons during an in-home visit, which each lesson lasting approximately 60 minutes. However, the control lessons focus solely on nutrition and the major food groups and do not include extensive content on infant-centered feeding. Paraprofessional instructors who provide the lessons for the control group families are different from the instructors who provide the HB lessons to prevent cross-contamination between the two groups.

### Procedures

Data are obtained by trained data collectors using various methods, including self-report questionnaires, anthropometric assessment, and videotaping of mealtime observations. All data collectors are trained to be as unobtrusive as possible when they are videotaping the mother-infant feeding interaction and to conduct data collections in an interactional style least likely to provoke social desirability characteristics on the part of the mother.

Data collection in both states occurs at baseline (prior to the first lesson), when the baby is 6 months old, and when the baby is 1 year old. The intervals for data collection were chosen to reflect points in time critical in determining outcomes, such as following the in-home learning and again, when the infant is 12 months old, determining sustainability. We expect that the follow-up (at 12 months of age) for mother-infant dyads during this critical developmental stage will be sufficient in measuring sustainability of change in maternal responsiveness, feeding style, and feeding practices.

To ensure consistency and accuracy of data collection and videotaping, a 2-day intensive training session is held for data collectors prior to the collection of data, followed by booster sessions in years 2 and 3. Training consists of a standardized training guide and protocol including review of the instruments, measurement of height/length and weight, and videotaping the mother-infant feeding interaction. Data collectors are monitored for quality and fidelity to the protocol on a quarterly basis. Weekly telephone contact and email communication also occur between the project manager and the data collector to facilitate open communication and fidelity to the protocol.

### Measures

#### Maternal outcomes

**Maternal responsiveness **is defined as the sensitive and accepting behaviors identified in a mother toward her infant. The Parent-Child Interaction Scale-Feeding (PCI-F) [15], formerly Nursing Child Assessment Feeding Scale (NCAFS) [15], consists of 76 binary (yes/no) scored observation items designed to assess the mother-infant interactions during a feeding interaction in the home. The PCI-F has well-documented reliability and validity; Cronbach's alphas for the subscales range from .60 to .83 [15]. The PCI-F has been used extensively in research studies globally. The feeding observation will be videotaped in the infant's natural setting with his mother.

**Feeding style **represents the mother's beliefs about and approach to controlling her infant's feeding behaviors. The Infant Feeding Styles Questionnaire (IFSQ) [16] is a maternal self-report instrument designed to measure maternal beliefs and feeding style behaviors. The IFSQ was pretested and validated with 150 African American first-time mothers with children ≤ 2 years of age. Reliabilities range from .75-.95 [16].

**Feeding practices **are maternal behaviors relating to what is fed and how often; they are essential for ensuring appropriate eating habits throughout childhood. The Infant Feeding Scale (IFS) is a 65-item food frequency scale we developed as an adaptation of the Harvard Service Food Frequency Scale for Children [17]. It consists of two subscales: (a) one identifies foods and beverages consumed by the infant, including number of times offered daily, average number of spoonfuls eaten, total food and liquid consumed in a 24-hour period, and frequency of foods eaten during a week; (b) the second consists of the feeding environment and states where the infant ate, what the infant was doing during mealtimes, whether the television was on, if the mother sat with the infant, what, if any, of the same foods did the mother eat, how frequently the infant was fed, when the infant was fed, and who fed the infant.

### Infant outcome

#### Infant growth pattern

Appropriate **i**nfant growth patterns are the rate of weight-for-length gain based on gender, age, type of feeding, and birth weight, such that an infant stays between the 5th and 84th percentile. Experts recently revised the terminology for childhood obesity, including infants, categorizing weight-for-length values above the 95th percentile as "overweight" and between the 85th and 95th percentile as "at-risk for overweight."

Infant growth pattern is the measurement of an infant's weight and length plotted over time using z scores. The infant's length and weight will be measured at all three times points to provide an objective measure of weight status. The infant is weighed in a clean diaper on an electronic scale, positioned in the center of the scale tray and weighed to the nearest half ounce. If the infant is too active, an alternative measurement technique is used where the parent stands on a scale and holds the infant. Infant length is measured in the recumbent position. These data will be entered into Epi Info V3.4.3 to calculate weight-for-length z scores. Digital scales will be calibrated at each home prior to weights being taken.

**Maternal knowledge and self-efficacy **are being assessed at all three time points using the Maternal Knowledge and Self-Efficacy scale we developed from a previous study [12] and adapted for this study. The self-report scale consists of 13 knowledge items (with an internal consistency of .73) and seven self-efficacy items (with an internal consistency of .94), takes approximately 5 minutes to complete, and has been used with lower-income mothers of infants [12].

### Maternal and infant conditions that may influence outcomes

Maternal depression and infant temperament are conditions that may impact the effectiveness of the intervention. Maternal and infant conditions will be assessed at the three time points. Maternal depression data will be collected using three questions from the Edinburgh Postnatal Depression Scale (EPDS-3) [18], which has an internal consistency of .8 [18]. Infant temperament will be assessed using the Revised Infant Behavior Questionnaire (IBQ-R) [19], a 47-item measure with internal reliability range of .73-.94. At baseline, we will also collect data on the mother's age, education, history of infant feeding, work status and occupation, ethnicity, marital status, number of adults in the home, and the presence of other adult caregivers (e.g., grandmother).

### Feasibility variables (process evaluation)

#### Mothers: recruitment, retention, and satisfaction

Participant eligibility information will be tracked by the supervisor for each county in both states for all participants approached and will be recorded on a recruitment chart and county recruitment form by the project manager (PM). The PM will track recruitment and attrition information for all participants enrolled in the study on a participant tracking form. Maternal satisfaction information will be assessed using the Maternal Satisfaction 5-item brief survey via a mailed-in postcard at the end of the lessons and by quarterly telephone calls to random participants made by an evaluator separate from the research team directly involved with delivering the intervention.

### Sample size, power calculations, and data analysis

#### Sample size and power calculations

A convenience sample of 394 mother-infant dyads (186 each per intervention and control group) is anticipated. Effect sizes were estimated from similar available data and are calculated to be conservative to avoid Type II errors. To assess differences in groups cross-sectionally, the maternal Nursing Child Assessment Satellite Training (NCAST) data were used with a reported an effect size *(d*) of .35. The minimal detectable effect size (MDES) with power of .80 with an alpha level of 5% for 2-tailed tests is .33 expressed as Cohen's d. Effect sizes exceeding .30 are deemed more likely to reach clinical significance. We used normative data from the NCAST database containing data from 431 African American women to provide examples of projected changes in outcomes that, if produced by the HB intervention, will be detected as statistically significant.

The MDES was calculated for the pair-wise group differences using an approach described by Rochon [20]. We used H3 type of hypothesis since the model contains group-by-time interaction, and the standardized MDES was approximately 0.4. That is, if the minimum difference between groups divided by the maximum standard deviation across two time points is equal to 0.4 or greater, then the treatment difference inconsistent over time will be detected as statistically significant with power .80 and alpha = .05 for 2-tailed tests. A 70% to 75% completion rate is typical for nutrition education. These factors have led us to propose a sample size for analysis by Time 3 of 278 mother-infant dyads, 139 per group after accounting for estimated attrition. Including oversampling to accommodate a conservative estimate of 25% attrition after 1 year, the final sample size to be enrolled is 372, 186 per group and per state (in each state, 93 mothers and infants will be recruited for each group).

##### Data analysis

Frequency distributions, measures of central tendency, and variability will be calculated for all variables of interest. The outcomes and maternal and infant conditions (e.g., maternal depression, weight, height, infant age, type of feeding) will be compared across groups at baseline using multivariate analysis of variance (MANOVA), chi-square tests, or Generalized Linear Mixed Effects Models (GLME) with Poisson or gamma distributed errors, if normalizing transformations are not successful for continuous variables. If differences between groups are observed despite computerized minimization, the relevant variables will be treated as covariates in our post-experiment analyses. The primary analysis is of the intent-to-treat type.

## Discussion

If this project is successful, the expected outcomes are that the intervention will be effective in helping mothers with young infants develop healthy infant feeding practices that will contribute to the overall health and development of the infant, and in reducing the risk of childhood obesity in vulnerable populations. If effective, the Healthy Babies project will result in a targeted baby-centered feeding support-education intervention targeting economically and educationally disadvantaged mothers of infants under the age of 1 year. Very few empirical studies have focused on early feeding interventions, yet many new parents, particularly lower-income parents, lack key knowledge about basic infant nutrition and feeding needs.

Our home visitation model refers to a structured model of interaction with families over a period of time. This type of home visitation is implemented as a primary prevention strategy. Cost-effectiveness of the HB intervention program will be considered in terms of intensity of services (e.g., number of visits) and qualifications and salary requirements of staff. One limitation to the study, however, is the use of convenience sampling, which may limit the generalizablity of the study findings to the target population. The "product" will be an intervention that can be used within community settings and is accessible, feasible, and accepted by the target population. The long-term goal of this intervention is to contribute to curbing the rising rates of childhood obesity through an effective multi-component, relationship skill-building, educational intervention that fosters infant-centered feeding to promote healthy maternal responsiveness, feeding styles, and feeding practices as infants transition to solid foods.

## List of Abbreviations

HB: Healthy Babies; RCT: randomized controlled trial; TPB: theory of planned behavior; USDA: United States Department of Agriculture; EFNEP: Expanded Food and Nutrition Education Program; WIC: Women, Infants and Children; NCAST: Nursing Child Assessment Satellite Training; MDES: minimal detectable effect size; PCI-F: Parent-Child Interaction Scale-Feeding; NCAFS: Nursing Child Assessment Feeding Scale; IFSQ: Infant Feeding Styles Questionnaire; TIFS: The Infant Feeding Scale; EPDS: Edinburgh Postnatal Depression Scale; IBQ-R: Revised Infant Behavior Questionnaire; PM: project manager; MANOVA: multivariate analysis of variance; GLME: Generalized Linear Mixed Effects Model; HBICF: Healthy Babies Infant-Centered Feeding.

## Competing interests

The authors declare that they have no competing interests.

## Authors' contributions

MAH, BLO, SB, HBH, GA, and LVE conceived the project, contributed to the development of the clinical trial and the procurement of the funding, participated in its design and coordination, and helped draft the manuscript. JL and LS assisted in the development and revision of the manuscript along with the study investigators. All authors read and approved the final manuscript.

## Pre-publication history

The pre-publication history for this paper can be accessed here:

http://www.biomedcentral.com/1471-2458/11/868/prepub

## References

[B1] Koplan JP Liverman CT Kraak VI (Eds.) Preventing childhood obesity health in the balance 2005 Washington, DC: Institute of Medicine National Academies Press 22379642

[B2] Kim J Peterson KE Scanlon KS Fitzmaurice GM Must A Oken E Rifas-Shiman L Rich-Edwards JW Gillman MW Trends in overweight from 1980 through 2001 among preschool-aged children enrolled in a health maintenance organization Obesity 2006 14 1107 1112 10.1038/oby.2006.126 16899790

[B3] National Center for Health Statistics NHANES data on the prevalence of overweight among children and adolescents: United States, 2003-2004 http://www.cdc.gov/nchs/products/pubs/pubd/hestats/overweight/overwght_child_03.htm

[B4] Goran MI Ball GDC Cruz ML Obesity and risk of type 2 diabetes and cardiovascular disease in children and adolescents Journal of Clinical Endocrinology and Metabolism 2003 88 1417 1427 10.1210/jc.2002-021442 12679416

[B5] Crocetti M Dudas R Krugman S Parental beliefs and practices regarding early introduction of solid foods to their children Clinical Pediatrics 2004 43 541 547 10.1177/000992280404300606 15248007

[B6] Horodynski MA Olson B Arndt MJ Brophy-Herb HE Shirer K Shemanski R Low-income mothers' decisions regarding when and why to introduce solid foods to their infants: influencing factors Journal of Community Health Nursing 2007 24 101 118 10.1080/07370010701316247 17563282

[B7] Farrow C Blissett J Does maternal control during feeding moderate early infant weight gain? Pediatrics 2006 118 e293 e298 10.1542/peds.2005-2919 16882774

[B8] Wen LM Baur LA Rissel C Wardle K Alperstein G Simpson JM Early intervention of multiple home visits to prevent childhood obesity in a disadvantaged population: a home-based randomised controlled trial (Healthy Beginnings Trial) BMC Public Health 2007 7 76 10.1186/1471-2458-7-76 17490492 PMC1877802

[B9] Horodynski M Olson B Brophy-Herb H Silk K Shirer K The infant feeding series (TIFS) curriculum Journal of Nutrition Education and Behavior 2008 40 187 188 10.1016/j.jneb.2007.03.004 18457788

[B10] Horodynski M Olson B Brophy-Herb H Shirer K Arndt MJ Silk K Delaying the introduction of solid food to infants: a six-lesson curriculum for community educators Journal of Nutrition Education and Behavior 2006 38 Suppl 1 16 17

[B11] Ajzen I Kuhl J, Beckmann J From decisions to actions: a theory of planned behavior Action-control: From Cognition to Behavior 1985 Heidelberg: Springer 11 39

[B12] Brophy-Herb HE Silk KJ Horodynski MA Olson B Mercer L Key theoretical frameworks for intervention: understanding and promoting behavior change in parent-infant feeding choices in a low-income population Journal of Primary Prevention 2007 30 191 208 19283484 10.1007/s10935-009-0169-9

[B13] Moher D Schulz KF Altman DG The CONSORT statement: revised recommendations for improving the quality of reports of parallel group randomized trials BMC Medical Research Methodology 2001 1 1191 4 10.1186/1471-2288-1-2 PMC32201 11336663

[B14] Mutch B McKay C Michigan's ES/WIC nutrition education initiative: breastfeeding peer counselor initiative (BFI) 1996 Michigan State University Extension

[B15] Sumner G Spietz A NCAST caregiver/parent-child interaction feeding manual 1994 Seattle, WA: NCAST Publications, University of Washington, School of Nursing

[B16] Borja JB Mendes MA Bentley MA Zimmer C Adair LS The Infant Care Risk of Obesity Study Group Development and validation of the Infant Feeding Style Questionnaire (IFSQ): a tool for measuring parental feeding styles related to infant nutritional status 2007 Manuscript in preparation

[B17] Suitor CW Gardner JD Development of an interactive, self-administered computerized food frequency questionnaire for use with low-income women Journal of Nutrition Education 1992 24 82 86

[B18] Kabir K Sheeder J Kelly SK Identifying postpartum depression: are 3 questions as good as 10? Pediatrics 2008 122 e696 e702 10.1542/peds.2007-1759 18762505

[B19] Gartstein MA Rothbart MK Studying infant temperament via the revised Infant Behavior Questionnaire Infant Behavior and Development 2003 26 64 86 10.1016/S0163-6383(02)00169-8

[B20] Rochon J Sample size calculations for two-group repeated-measures experiments Biometrics 1991 47 1383 1398 10.2307/2532393

